# Lung inflammation by fungus, *Bjerkandera adusta* isolated from Asian sand dust (ASD) aerosol and enhancement of ovalbumin-induced lung eosinophilia by ASD and the fungus in mice

**DOI:** 10.1186/1710-1492-10-10

**Published:** 2014-02-05

**Authors:** Boying Liu, Takamichi Ichinose, Miao He, Fumihisa Kobayashi, Teruya Maki, Seiichi Yoshida, Yasuhiro Yoshida, Keiichi Arashidani, Hirohisa Takano, Masataka Nishikawa, Guifan Sun, Takayuki Shibamoto

**Affiliations:** 1 Environment and Chronic Non-communicable Disease Research Center, College of Public Health, China Medical University, 11001 Shenyang, China; 2 Department of Health Sciences, Oita University of Nursing and Health Sciences, 870-1201 Oita, Japan; 3 College of Science and Engineering, Kanazawa University, 920-1192 Ishikawa, Japan; 4 Department of Immunology and Parasitology, School of Medicine, University of Occupational and Environmental Health, Japan, 807-8555 Fukuoka, Japan; 5 Environmental Health Division, Department of Environmental Engineering, Graduate School of Engineering, Kyoto University, 615-8530 Kyoto, Japan; 6 Environmental Chemistry Division, National Institute for Environmental Studies, 305-8506 Ibaraki, Japan; 7 Department of Environmental Toxicology, University of California, Davis, CA 95616, USA; 8 Present address: Department of Prophylaxis and Health Care, The Fourth Affiliated Hospital of China Medical University, 110032 Shenyang, China

**Keywords:** Asian sand dust, *Bjerkandera adusta*, Fungus, Lung eosinophilia, Asthma

## Abstract

**Background:**

*Bjerkandera adusta* (*B. adusta*) is one of the most important etiological fungi associated with chronic cough. However, precise details of the inflammatory response to exposure are not well understood yet. *B. adusta* was recently identified in Asian sand dust (ASD) aerosol. Therefore, in the present study the exacerbating effects of ASD on *B. adusta*-induced lung inflammation and *B. adusta* + ASD on ovalbumin (OVA)-induced murine lung eosinophilia were investigated using experimental mice.

**Methods:**

In order to prepare testing samples, *B. adusta* obtained from ASD aerosol was inactivated by formalin and ASD collected from the atmosphere was heated to remove toxic organic substances (H-ASD). CD-1 mice were instilled intratracheally with 12 different samples prepared with various combinations of *B. adusta,* H-ASD, and OVA in a normal saline solution. The lung pathology, cytological profiles in bronchoalveolar lavage fluid (BALF), and the levels of inflammatory cytokines/chemokines in BALF were investigated.

**Results:**

H-ASD aggravated the lung eosinophilia induced by *B. adusta* alone, which also aggravated the lung eosinophilia induced by OVA. The mixture of OVA, H-ASD, and *B. adusta* caused serious fibrous thickening of the subepithelial layer, eosinophil infiltration, and proliferation of goblet cells in the airways along with remarkable increases of IL-13, eotaxin, IL-5, and MCP-3 in BALF.

**Conclusions:**

The results of the present study demonstrated that *B. adusta* isolated from ASD aerosol induces allergic lung diseases. H-ASD enhanced allergic reactions caused by OVA or *B. adusta*. A mixture of *B. adusta*, H-ASD, and OVA caused the most remarkable exacerbation to the allergic airway inflammation via remarkable increases of pro-inflammatory mediators.

## Background

*Bjerkandera adusta* (*B. adusta*) is known to occur throughout the world. This fungus produces abundant asexual spores, so-called arthroconidia, from the hyphae [1]. The asexual spores are known to be a fungal aeroallergen, which causes an allergic fungal cough [2]. Therefore, this fungus is one of the most significant etiological fungus-associated chronic cough (FACC) [1]. However, it is not well understood yet what type of inflammatory response is initiated in the lungs of human with exposure to components in *B. adusta* or infection caused by *B. adusta*.

Asian sand dust (ASD) storms arise from the dry and semi-arid areas of southern Mongolia and Northeast China. ASD is transported to East China, the Korean Peninsula, and Japan. It even travels across the Pacific Ocean to reach the United States [3,4]. Recently, *B. adusta* was identified in ASD aerosol, which was collected from the atmosphere in Suzu, Japan during the occurrence of an Asian dust event [5]. Many patients with FACC have been reported in Kanazawa (near Suzu City) [1]. Above all, ASD events have been known to exacerbate asthma for adults and children in Japan [6,7]. However, the role of ASD events in the occurrence of these diseases (FACC, asthma) is not well understood yet. It is crucial to investigate the effects of *B. adusta* and ASD on airway diseases in order to find the relevant treatment for patients of FACC or asthma.

In the present study, the exacerbating effects of ASD on *B. adusta*-induced lung inflammation and fungus and/or ASD on ovalbumin (OVA)-induced lung eosinophilia were investigated using a mouse model of asthma.

## Materials and methods

### Animals

A total of 168 male CD-1 mice (5 weeks of age) were purchased from Charles River Japan Inc. (Kanagawa, Japan). The abnormal or unhealthy mice were removed after one week and the remaining healthy mice were used at 6 weeks of age. The mice were fed commercial diet CE-2 (CLEA Japan Inc., Tokyo, Japan) and given water *ad libitum*. The mice were housed in plastic cages lined with soft wood chips. The cages were placed in a conventional room, which was air conditioned at 23°C and 55–70% humidity with a light/dark (12 h/12 h) cycle. CD-1 male mice were used because of their moderate responsiveness to airway inflammation caused by OVA [8]. The present study adhered to the U.S. National Institutes of Health guidelines for the use of experimental animals. The animal care method was approved by the Animal Care and Use Committee at Oita University of Nursing and Health Sciences, Oita, Japan.

### Preparation of particle samples

ASD was collected from the atmosphere using a high-volume air sampler with a Teflon filter at the University of Occupational and Environmental Health, Kitakyushu, Fukuoka, Japan during May 1^st^– 3^nd^, 2011 right after a massive 3-day dust storm event occurred in East Asia. The flow rate for collection was 770 L/min. The instrumental classification size (defined as the 50% cut-off size of the aerodynamic diameter) was 5.9 μm in the 1st stage, 2.8 μm in the 2nd stage, 1.7 μm in the 3rd stage, 0.91 μm in the 4th stage, and < 0.91 μm of back-up. The size distribution peak of ASD was at 3.8 μm. The density ranges of the ambient particulate matter measured by LIDAR (Light Detection and Ranging) on May 1^st^ - 3^rd^, 2011 were 350–550 μg/m^3^ in Nagasaki (Nagasaki Prefectural Institute of Public Health and Environmental Science), Japan.

The particle samples in a glass bottle were heated at 360°C for 30 min in an electric heater to remove toxic materials (microbiological materials, sulfate, nitrate, etc.) and labelled as H-ASD.

### Fungus preparation

The aerosol samples—collected at 400 m above the ground using a tethered balloon equipped with an aerosol sampler on May 7, 2008 in Suzu, Ishikawa Prefecture—were inoculated onto four culture agar plates (nutrient agar, blood agar, potato dextrose agar, and Sabouraud dextrose agar). After a few days of incubation, several microbial colonies on the agar plates were transferred to new agar plates. A total of 8 isolates were obtained. The 18S rRNA gene (ca. 1650 bp) and the internal transcribed spacer (ITS) region (ca. 620 bp) of the fungal isolate were sequenced and compared with known sequences from the DDBJ database. Sequences of the isolate were the most closely related to *B. adusta* (similarities: > 99.9%). The fungal isolate was also confirmed as *B. adusta* by microscopic observation [5].

The density ranges of the asexual spore (4-5 μm size) in the atmosphere of Suzu City during dust storm event (May 1^st^ -3^rd^, 2011) were about 1 million – 6 million particles/m^3^, which were remarkably higher than the general levels (about 5000 particles/m^3^) [9]. The asexual spores may cause adverse effect to the health. Because *B. adusta* produces asexual spores from the hyphae, the fungal hyphae were used in this study.

The fungal hyphae cultivated were inactivated with 1% formalin for 1 day at 4°C according to the vaccine manufacture method with slight modifications [10]. Briefly, the inactivated *B. adusta* solution was centrifuged at 1200 rpm for 10 min. The resulting pellet was suspended in a normal saline solution (Otsuka Co., Kyoto, Japan) and then the suspension was sonicated for 1 min with a UD-201 type ultrasonic disrupter with micro tip (Tomy, Tokyo, Japan) under cooling conditions. The suspension was centrifuged at 12,000 rpm for 10 min 5 times using a normal saline solution. The resulting supernatant was removed and the residual materials were dried under reduced pressure.

### Analysis of LPS and β-glucan in H-ASD and *B. adusta*

The contents of LPS and β-glucan in H-ASD and *B. adusta* were measured by a kinetic assay using Endospec ES-24S set (Seikagaku Corp., Tokyo, Japan) for LPS activity and Glucatell Kit (Associates of Cape Cod. Inc., MA, USA) for β-glucan activity. The experiments were performed according to the manuals provided by the manufacturers. Briefly, approximately 5 mg of H-ASD or 10 μg of *B. adusta* (dry weight) was suspended in 1 mL water (LPS and β-glucan free; Seikagaku Corp., Tokyo, Japan) and then allowed to stand at room temperature for 2 h. LPS and β-glucan concentrations in the supernatants were determined using a Pyro Color-MP:Chromogenic Diazo-Coupling Kit (Associates of Cape Cod. Inc., MA, USA). The detection limits for LPS and β-glucan were 0.001 EU/ml and 2 pg/ml, respectively.

### Study protocol

Experimental mice were divided into twelve groups (n = 14 per group) and each group was treated with a specific testing sample. The 12 testing samples (0.1 mL each of 0.9% NaCl normal saline solution) prepared for the present study were control (containing normal saline alone); H-ASD (0.1 mg H-ASD alone); *B.ad* 2 (2 μg *B. adusta* alone); *B.ad* 8 (8 μg *B. adusta* alone); H-ASD + *B.ad* 2 (0.1 mg H-ASD and 2 μg *B. adusta*); H-ASD + *B.ad* 8 (0.1 mg H-ASD and 8 μg *B. adusta*); OVA alone (1 μg); OVA + H-ASD (1 μg OVA and 0.1 mg H-ASD); OVA + *B.ad* 2 (1 μg OVA and 2 μg *B. adusta*); OVA + *B.ad* 8 (1 μg OVA and 8 μg *B. adusta*); OVA + H-ASD + *B.ad* 2 (1 μg OVA, 0.1 mg H-ASD, and 2 μg *B. adusta*); and OVA + H-ASD + *B.ad* 8 (1 μg OVA, 0.1 mg H-ASD, and 8 μg *B. adusta*). One previous report indicated that the microbe was present in ASD aerosol at approximately 10% [11]. The instillation dose of *B. adust* was set at 2% (2 μg) and 8% (8 μg) for the one-time ASD instillation dose (0.1 mg), because one kind of microbial species was not considered to occupy 10% of total microbe in ASD aerosol. The one-time ASD instillation dose (0.1 mg) was conducted according to a previously reported method [12].

*B. adusta,* H-ASD, and OVA were suspended in a normal saline solution (Otsuka Co., Kyoto, Japan) and sonicated for 5 min with ultrasonic disrupter Model UD-201 with micro tip (Tomy, Tokyo, Japan) under cooling conditions. A 0.1 mL saline solution containing the samples described above was intratracheally instilled to the mice through a polyethylene tube under anesthesia with 4% halothane (Takeda Chemical, Osaka, Japan) four times at 2-week intervals. One day after the last administration, the mice from all groups were killed by exsanguination under deep anesthesia by intraperitoneal injection of pentobarbital.

The mice in each group (14 mice) were divided into two sub-groups. The first (6 mice) was used for pathological evaluation. The remaining 8 mice were used for the analysis of cytokine and chemokine in the bronchoalveolar lavage fluids (BALFs).

### Pathological evaluation and BALF preparation

The lungs were fixed by a 10% neutral phosphate-buffered formalin solution. After separation of the lobes, blocks (2 mm thickness) were taken for paraffin embedding. The embedded blocks were sectioned (3 μm thickness) and then stained with periodic acid-schiff (PAS) to evaluate the degree of proliferation of goblet cells in the bronchial epithelium and thickening of the subepithelial layer in the main bronchus. The sections were also stained with hematoxylin and eosin (H & E) in order to evaluate the degree of infiltration of eosinophils or lymphocytes in the airway from proximal to distal [12,13].

A pathological analysis of the inflammatory cells and epithelial cells in the airways of each lung lobe on the slides was performed using a Nikon ECLIPSE light microscope (Nikon Co., Tokyo, Japan). The degree of proliferation of goblet cells in the bronchial epithelium was graded on the following scale: 0, not present; 1, slight; 2, mild; 3, moderate; 4, moderate to marked; and 5, marked. ‘Slight’ was defined as less than 20% of the airway having goblet cells stained with PAS; ‘mild’ as 21 –4 0%; ‘moderate’ as 41 – 60%; ‘moderate to marked’ as 61–80%; and ‘marked’ as more than 81% [12,13]. The degree of thickening of the subepithelial layer in the main bronchus was graded on the following scale: 0, not present; 1, slight; 2, mild; 3, moderate; 4, moderate to marked; and 5, marked. ‘Slight’ was defined as 5–12 μm of the main bronchus with fibroblasts stained with PAS; ‘mild’ as 13–20 μm; ‘moderate’ as 21–28 μm; ‘moderate to marked’ as 29-36 μm; and ‘marked’ as more than 37 μm [13].

Pathological changes were assessed on one slide stained with PAS per mouse. This evaluation procedure was performed by two pathologists who cross-checked the data with blinded specimens. The values were mean ± SE (n = 6).

BALF preparation and cell counts were conducted according to a previously reported method [12,13]. The total amount of the lavages collected from individual mice was measured for the protein levels of cytokines and chemokines in BALF. The total cell counts of each fresh fluid specimen were determined using a hemocytometer.

### Quantitation of cytokines and chemokines in BALFs

The cytokine protein levels in the BALF were determined using ELISA. IL-5 and IL-12 were measured using an ELISA kit from Thermo Scientific (Rockford, IL, USA). MCP-3 was measured using an ELISA kit from Bioscience (San Diego, CA, USA). KC, MCP-1, MIP-1α, RANTES, TNF-α, Eotaxin, IFN-γ, TGF-β1, IL-1β, IL-4, IL-6, IL-10, IL-13, and IL-33 were measured with an ELISA kit from R&D Systems (Minneapolis, MN). These cytokines and chemokines were measured by individual ELISA kit.

### Measurement of antigen-specific IgG1 and IgE antibodies

OVA-specific immunoglobulin E (IgE) and IgG1 antibodies were measured using a Mouse OVA-IgE ELISA kit and a Mouse OVA-IgG1 ELISA kit (Shibayagi, Shibukawa, Japan). The absorption at 450 nm (sub-wavelength, 620 nm) for OVA-specific IgE and IgG1 antibody was measured using a microplate reader (Spectrafluor; Tecan, Salzburg, Austria).

### Statistical analysis

Statistical analyses of the pathologic evaluations of the airway, cytokine and chemokine proteins in BALF were conducted using the Tukey Test for Pairwise Comparisons (KyPlot Ver.5, Kyens Lab Inc., Tokyo, Japan). Differences among groups were determined as statistically significant at a level of p < 0.05.

## Results

### Contents of LPS and β-glucan in H-ASD and *B. adusta*

LPS and β-glucan were not detected in the H-ASD samples. The β-glucan content in *B. adusta* was 32.8 ng/mg. LPS was not detected in *B. adusta.*

### Enhancement of cell numbers in BALF by *B. adusta* and H-ASD

**Figure 1:**
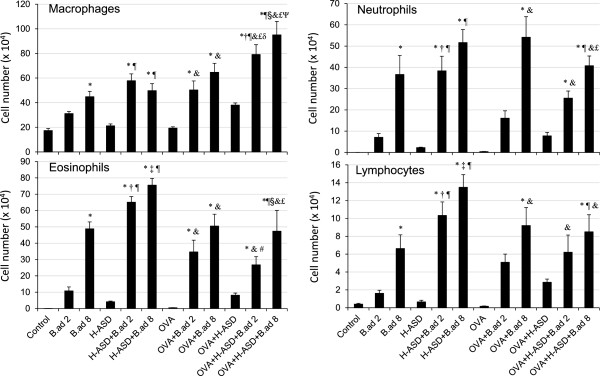
**Cellular profile in bronchoalveolarlavage fluids (BALF).** All values expressed as mean ± SE. ^*^p < 0.05 vs. Control; ^†^*P* < 0.05 vs. *B.ad* 2; ^‡^p < 0.05 vs. *B.ad* 8; ^¶^p < 0.05 vs. H-ASD; ^#^p < 0.05 vs. H-ASD + *B.ad* 2; ^§^p < 0.05 vs. H-ASD + *B.ad* 8; &p < 0.05 vs. OVA; ^£^p < 0.05 vs. OVA + H-ASD; ^δ^p < 0.05 vs. OVA + *B.ad* 2; ^ψ^p < 0.05 vs. OVA + *B.ad* 8.

Figure 1 shows the numbers of macrophages, neutrophils, eosinophils, and lymphocytes determined in BALF from the control and treated groups. The significant increase of these BAL cells was resulted by *B.ad* 8 alone, H-ASD **+** *B.ad* 2/*B.ad* 8, OVA + *B.ad* 2/*B.ad* 8, and OVA + H-ASD + *B.ad* 2/*B.ad* 8. Numbers of BAL cells, especially neutrophils and eosinophils of all substances increased dose relatively by *B. adusta*. In particular, significant effects by the addition of *B.ad* 8 were observed in neutrophils and eosinophils. Overall, H-ASD played an important role in the enhancement of inflammatory cells in BALF.

On the other hand, OVA + H-ASD + *B.ad* 2 and OVA + H-ASD + *B.ad* 8 significantly decreased eosinophil numbers compared with H-ASD + *B.ad* 2 and H-ASD + *B.ad* 8, suggesting that OVA inhibits the action of *B. adusta* toward eosinophil in BALF.

### Enhancement of pathologic changes in the airway by H-ASD and *B. adusta*

**Figure 2:**
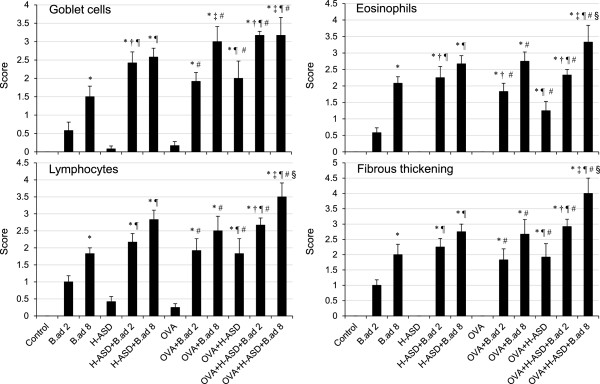
**Evaluation of pathological changes in the murine airway.** The degree of pathological changes in the airway was estimated as: (0) none; (1) slight; (2) mild; (3) moderate; (4) moderate to marked; (5) marked. All values were expressed as mean ± SE (n = 6). Statistical analyses were conducted using Tukey for Pairwise Comparisons. ^*^p < 0.05 vs. Control; ^†^*P* < 0.05 vs. *B.ad* 2; ^‡^p < 0.05 vs. *B.ad* 8; ^¶^p < 0.05 vs. H-ASD; ^#^p < 0.05 vs. OVA; ^§^p < 0.05 vs. OVA + H-ASD.

**Figure 3:**
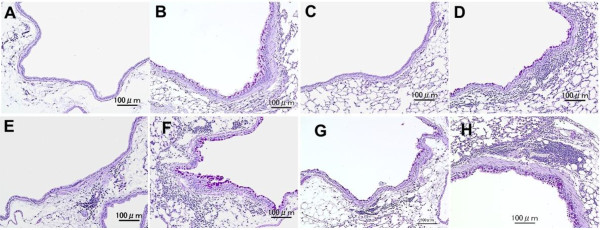
**Effects of testing samples on pathological changes in the lungs.** No pathologic alterations were seen in the lungs of the control **(A)**. Mild proliferation of goblet cells that have mucus stained pink with PAS in airway epithelium and moderate fibrous thickening of the subepithelial layer in the airway exposed to *B.ad* 8 alone **(B)**. Very slight proliferation of bronchial cells exposed to H-ASD alone **(C)**. Mild proliferation of goblet cells that have mucus stained pink with PAS in the airway epithelium and infiltration of inflammatory cells in the submucosa of airway and mild fibrous thickening of the subepithelial layer in the airway exposed to H-ASD + *B.ad* 8 **(D)**. Very slight proliferation of goblet cells in the airway epithelium and slight infiltration of inflammatory cells in the submucosa of airway exposed to OVA alone **(E)**. Mild to moderate proliferation of goblet cells in the airway epithelium and infiltration of inflammatory cells in the submucosa of airway along with mild fibrous thickening of the subepithelial layer in the airway exposed to OVA + *B.ad* 8 **(F)**. Slight goblet cell proliferation and slight infiltration of inflammatory cells in the submucosa of airway exposed to OVA + H-ASD **(G)**. Moderate goblet cell proliferation, numerous inflammatory cells in the submucosa of airway, and serious fibrous thickening of the subepithelial layer in the airway and macrophages scattered in alveoli exposed to OVA + H-ASD + *B.ad* 8 **(H). (A–H)** PAS stain; bar = 100 μm.

**Figure 4:**
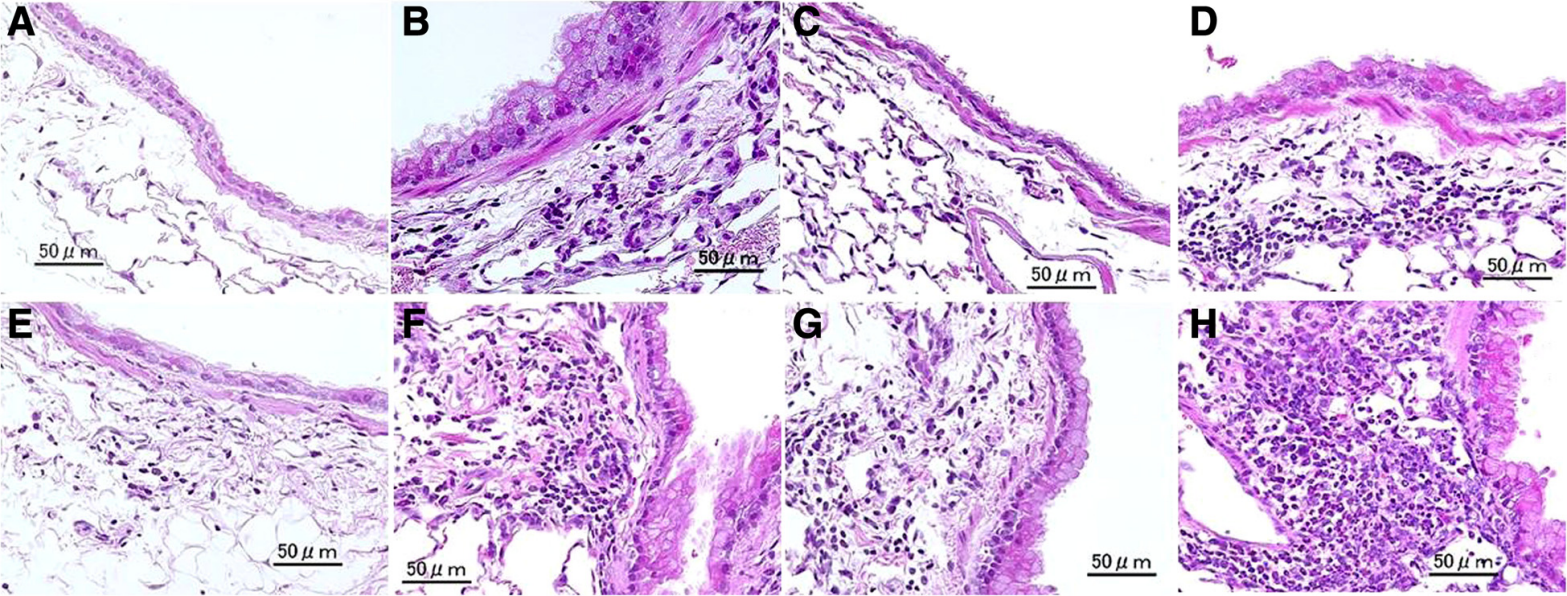
**Effects of *B. adusta *on infiltration of inflammatory cells in the airway.** No pathologic changes in the lungs of the control **(A)**. Slight infiltration of eosinophils and lymphocyte in the submucosa of airway exposed to *B.ad* 8 alone **(B)**. No pathologic changes in the airway and in the alveoli exposed to H-ASD alone **(C)** H-ASD + *B.ad* 2 caused mild accumulation of lymphocytes in the submucosa of airway **(C)**. Mild to moderate eosinophils, neutrophils and lymphocytes in the submucosa of airway exposed to H-ASD + *B.ad* 8 **(D)**. Very slight infiltration of eosinophil and lymphocytes in the submucosa in the airway **(E)**. Mild to moderate infiltration of eosinophils, neutrophils and lymphocytes in the submucosa of airway exposed to OVA + *B.ad* 8 **(F)**. Mild infiltration of eosinophils, neutrophils and lymphocytes in the submucosa of airway exposed to OVA + H-ASD **(G)**. Marked infiltration of eosinophils, neutrophils and lymphocytes in the submucosa of airway exposed to OVA + H-ASD + *B.ad* 8 **(H)**. **(A–H)** HE stain; bar = 50 μm.

Figure 2 shows the pathological changes caused by the particle samples in the murine airway. Figures 3 (PAS stain) and 4 (HE stain) show the effects of H-ASD and *B. adusta* on pathological changes in the lungs. No pathologic alteration was observed in the lungs of the control (A in Figures 3 and 4). Effects by particle samples exhibited results similar to the case of cell numbers in BALF. Exposure to *B.ad* 8 alone (B in Figure 3) resulted in a significant increase in proliferation of goblet cells, infiltration of eosinophils, and lymphocytes in the submucosa of the airway compared with the control (refer to Figure 2), and caused fibrous thickening of the subepithelial layer in the airway (B in Figure 3). H-ASD alone (C in Figures 3 and 4) or OVA alone (E in Figures 3 and 4) caused only slight proliferation of bronchial cells and infiltration of macrophages in the alveoli but no change of infiltration of eosinophils.

When *B.ad* 2 or *B.ad* 8 was mixed with H-ASD, proliferation of goblet cells in the airway epithelium and infiltration of eosinophils and lymphocytes in the submucosa of the airway and fibrous thickening of the subepithelial layer in the airway were observed (D in Figures 3 and 4). These pathological changes in the H-ASD + *B.ad* 8 group were greater than in the H-ASD + *B.ad* 2 group (Figure 2).

When *B.ad* 2 was added to OVA, mild proliferation of goblet cells in the airway epithelium and infiltration of eosinophil and lymphocytes in the submucosa along with mild fibrous thickening of the subepithelial layer in the airway were recognized. A further increase of these pathological changes (F in Figures 3 and 4) was observed by the addition of *B.ad* 8 compared with OVA + *B.ad* 2. These changes were significant compared with the control and OVA alone (Figure 2).

OVA + H-ASD caused mild pathological alterations (G in Figures 3 and 4), whereas the addition of *B.ad* 2 increased pathological changes such as mild to moderate goblet cell proliferation, eosinophil infiltration, and accumulation of lymphocyte in the submucosa of airway compared with the control, *B.ad* 2, H-ASD, and OVA. As shown in Figure 2, the addition of *B.ad* 8 caused the greatest increase of four items in pathological changes (H in Figures 3 and 4) compared with the control and promoted a serious fibrous thickening in the airway (H in Figure 3). Overall, H-ASD played an important role in the enhancement of pathological changes.

### Enhancement of cytokines and chemokines in BALF by particle samples

**Figure 5:**
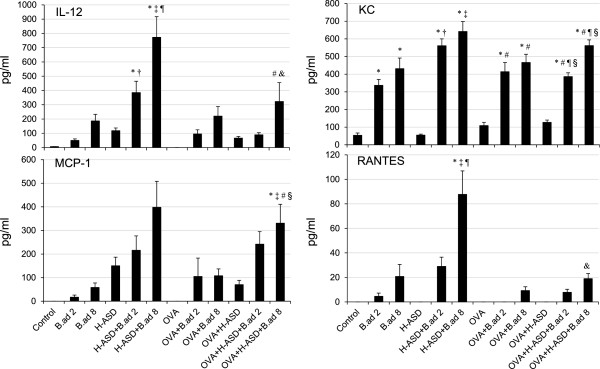
**Expressions of IL-12, KC, MCP-1 and RANTES in BALF.** All values were expressed as mean ± SE (n = 8). ^*^p < 0.05 vs. Control; ^†^*P* < 0.05 vs. *B.ad* 2; ^‡^p < 0.05 vs. *B.ad* 8; ^¶^p < 0.05 vs. H-ASD; ^#^p < 0.05 vs. OVA; ^§^p < 0.05 vs. OVA + H-ASD; ^&^p < 0.05 vs. H-ASD + *B.ad* 8.

**Figure 6:**
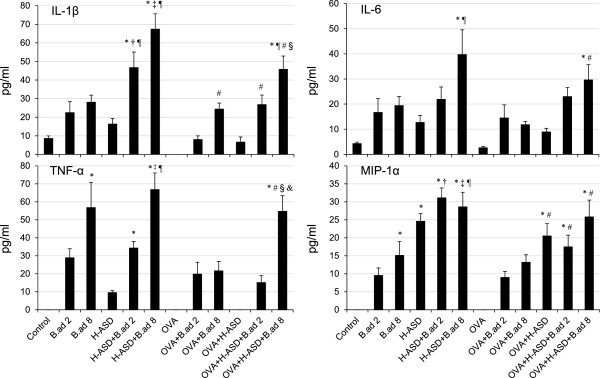
**Expressions of IL-1β, IL-6, TNF-α and MIP-1α in BALF.** All values were expressed as mean ± SE (n = 8). ^*^p < 0.05 vs. Control; ^†^*P* < 0.05 vs. *B.ad* 2; ^‡^p < 0.05 vs. *B.ad* 8; ^¶^p < 0.05 vs. H-ASD; ^#^p < 0.05 vs. OVA; ^§^p < 0.05 vs. OVA + H-ASD; ^&^p < 0.05 vs. OVA + *B.ad* 8.

**Figure 7:**
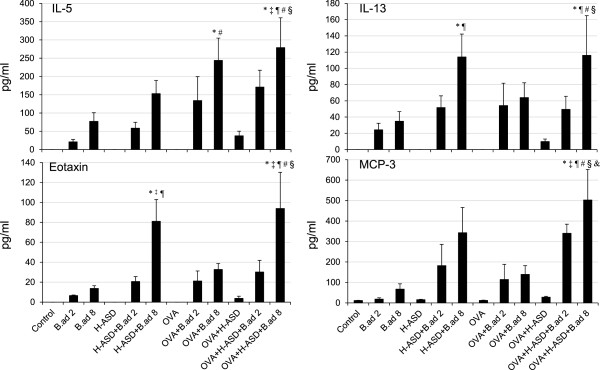
**Expressions of IL-5, IL-13, Eotaxin and MCP-3 in BALF.** All values were expressed as mean ± SE (n = 8). ^*^p < 0.05 vs. Control; ^†^*P* < 0.05 vs. *B.ad* 2; ^‡^p < 0.05 vs. *B.ad* 8; ^¶^p < 0.05 vs. H-ASD; ^#^p < 0.05 vs. OVA; ^§^p < 0.05 vs. OVA + H-ASD; ^&^p < 0.05 vs. OVA + *B.ad* 8.

**Figure 8:**
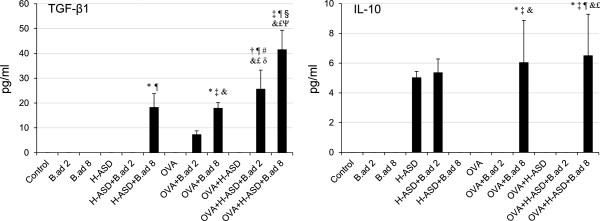
**Expressions of TGF-β1 and IL-10 in BALF.** All values were expressed as mean ± SE (n = 8). ^*^p < 0.05 vs. Control; ^†^*P* < 0.05 vs. *B.ad* 2; ^‡^p < 0.05 vs. *B.ad* 8; ^¶^p < 0.05 vs. H-ASD; ^#^p < 0.05 vs. H-ASD + *B.ad* 2; ^§^p < 0.05 vs. H-ASD + *B.ad* 8; &p < 0.05 vs. OVA; ^£^p < 0.05 vs. OVA + H-ASD; ^δ^p < 0.05 vs. OVA + *B.ad* 2; ^ψ^p < 0.05 vs. OVA + *B.ad* 8.

The protein levels of cytokines and chemokines determined in BALF are shown in Figures 5, 6, 7 and 8. As shown in Figure 5, *B. adusta* increased all proteins, in particular KC, determined in BALF with a dose relation. The mixture of H-ASD and *B. adusta* increased all proteins significantly. In particular, IL-12 increased 7-fold with the addition of *B.ad* 8 to H-ASD. A considerable increase of RANTES was also observed in this sample. In the case of the OVA treated groups, the addition of *B. adusta* also promoted an increase of these proteins, in particular KC. When *B.ad* 8 was added to an OVA + H-ASD sample, IL-12 and MCP-1 increased remarkably compared with samples treated with OVA alone. On the other hand, addition of OVA to H-ASD + *B. adusta* sample decreased IL-12, MCP-1 and RANTES, suggesting that OVA inhibits their activity.

Figure 6 shows the expression of IL-1β, IL-6, TNF-α, and MIP-1α in BALF. OVA alone did not exhibit any appreciable activity. H-ASD showed moderate effects of an increase of proteins, of which MIP-1α was the most affected. An increase of MIP-1α was also observed in the case of OVA + H-ASD. Obvious increases of all proteins with a dose relation were obtained by the presence of *B. adusta.* It is obvious that *B. adusta* plays an important role in the increase of these proteins. In particular, *B.ad* 8 alone clearly increased IL-1β and TNF-α compared with the control group. Overall, the addition of *B. adusta* to H-ASD and OVA samples increased proteins markedly. Generally, the presence of OVA decreased proteins. For example, the addition of OVA to H-ASD removed TNF-α completely.

Figure 7 shows the expression of IL-5, IL-13, Eotaxin, and MCP-3 in BALF. Only trace levels of MCP-3 were detected in the control groups. H-ASD and OVA alone did not increase any proteins analyzed in BALF. *B. adusta* alone increased levels of proteins slightly. On the other hand, a mixture of H-ASD or OVA with *B. adusta* clearly increased the levels of proteins. H-ASD + *B.ad* 8 notably increased IL-13 and Eotaxin compared with the control and H-ASD alone. In the OVA treated groups, OVA + *B.ad* 8 considerably increased IL-5 compared to the control and OVA-only groups. OVA + H-ASD + *B. adusta* markedly increased all proteins with a dose relation compared with all other samples.

Figure 8 shows the expression of TGF-β1 and IL-10 in BALF. No proteins were detected in the samples from control, *B. adusta* alone, OVA alone, and OVA + H-ASD. An obvious increase of TGF-β1 was observed in the BALF from the groups treated with mixtures of H-ASD and *B.ad* 8. In the OVA treated groups, the addition of *B.ad* 8 increased TGF-β1 and IL-10 moderately compared with the groups treated with OVA alone. OVA + H-ASD + *B. adusta* markedly increased TGF-β1 with a dose relation compared with all other samples. The significant increase of IFN-γ, IL-4 and IL-33 was not detected in this study.

**Figure 9:**
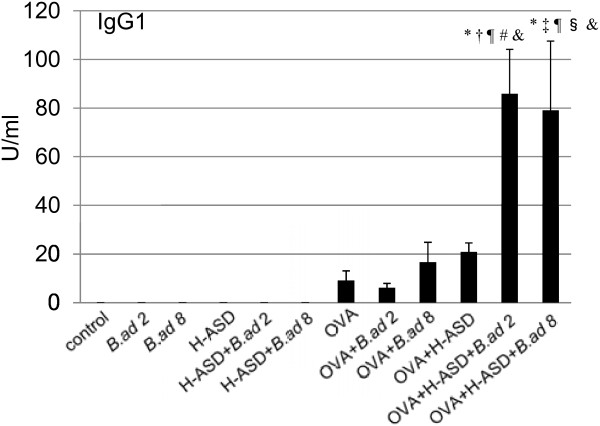
**Effects of testing samples on IgG1 production in serum.** According to the manufacturer’s protocol, 1 U of the anti-OVA IgG1 is defined as 160 ng of the antibody. All values were expressed as mean ± SE. ^*^p < 0.05 vs. Control; ^†^p < 0.05 vs. *B.ad* 2; ^‡^p < 0.05 vs. *B.ad* 8; ^¶^p < 0.05 vs. H-ASD; ^#^p < 0.05 vs. H-ASD + *B.ad* 2; ^§^p < 0.05 vs. H-ASD + *B.ad* 8; ^&^p < 0.01 vs. OVA.

Figure 9 shows the expression of OVA-specific IgG1 in serum. OVA + H-ASD + *B.ad* 2/*B.ad* 8 increased IgG1 compared with OVA alone. However, no induction of OVA-specific IgE was observed in any of these groups.

## Discussion

It is known that ASD aerosol contains many kinds of microorganisms [5,14]. *B. adusta,* isolated from the wind-borne ASD aerosol, may affect FACC or asthmatic patients negatively. In the present study, the exacerbating effects of ASD on *B. adusta*-induced lung inflammation and the exacerbating effects of the fungus and ASD on ovalbumin (OVA)-induced lung eosinophilia in a mouse model of asthma were examined. *B. adusta* is a mushroom fungus that grows in fields and colonizes rotting wood. The fungus, white-rot fungus, secretes multiple lignin peroxidase isozymes [15,16] and has been applied for the biodegradation of polycyclic aromatic hydrocarbons [17], synthetic dyes [18], and synthetic polymer nylon [19] in agricultural fields.

This study demonstrated that the formalin-inactivated fungus causes lung eosinophilia in mice. The inactivated *B. adusta* alone dose relatively increased neutrophils and eosinophils along with proinflammatory mediators MIP-1α, KC and TNF-α in BALF. Pathologically, the fungus caused eosinophil recruitment in the submucosa of the airway along with proliferation of goblet cells in the bronchial epithelium, suggesting that it acts as an antigen for human asthma. The treatment of inactivated *B.adusta* with H-ASD caused further increases in inflammatory cell numbers, pro-inflammatory cytokines (IL-1β, IL-5, IL-6, IL-12, IL-13), and chemokines (Eotaxin, RANTES, MIP-1α, KC, MPC-1, MCP-3) in BALF. The pathological changes in the airway induced by the co-treatment were more remarkable than *B. adusta* treatment alone, suggesting that H-ASD enhances *B.adusta*-induced lung eosinophilia via remarkable increases of these pro-inflammatory mediators, especially eosinophil-relevant chemokine MCP-3, cytokine IL-5 and Th2-mediated cytokine IL-13. Eosinophils are reportedly implicated in tissue destruction in allergic asthma [20]. Because, toxic eosinophil–derived proteins cause bronchial mucosal damage in asthmatic airways, they may exacerbate the symptoms of asthma [21]. IL-13 has been shown to stimulate B cells and subsequently produce antigen specific antibodies [22]. It also promotes mucous secretion and the production of mucous cells, such as goblet cells, in the bronchial epithelium [23]. Therefore, the airway injury resulting from co-treatment may be due to enhancement of eosinophilic airway-inflammation.

The pathway related to NF-κB activation by β-glucan reportedly indicated an association between TLR2 and Dectin-1 [24]. In fact, the presence of trace β-glucan (32.8 ng/mg) in *B. adusta* was detected using a kinetic assay in the present study. Therefore, it is proposed that the aggravation by H-ASD is due to the activation of TLR2-Dectin1-NF-κB signaling pathway that subsequently causes the production of inflammatory cytokines. However, the present study does not provide sufficient evidence to determine the role of β-glucan in allergic reactions.

In the case of the OVA treated groups, the addition of *B. adusta* increased eosinophil numbers along with their relevant cytokines and chemokines (Figure 7). Similar results were observed for neutrophil numbers and their relevant pro-inflammatory mediators (Figure 5). The asthma-like alterations induced in the airway by *B. adusta* with OVA were much higher than by treatment with *B. adusta* alone. Previous reports have demonstrated that co-treatment of TLR2-ligand Pam3Cys and OVA activates an OVA-associated Th2-biased immune response in experimental asthma [25]. These results suggest that *B. adusta* with β-glucan activates an OVA-related Th2-biased immune response through TLR2-dependent signaling pathways. The addition of H-ASD also enhanced Th2-biased immune response in the present study. The results are consistent with a previous study [14]. When mice were treated with OVA + H-ASD + *B. adusta*, serious fibrous thickening of the subepithelial layer, eosinophil infiltration, and proliferation of goblet cells in the airway along with remarkable increases of Th2 cytokine IL-13 and eosinophil relevant cytokines and chemokines induced in BALF were observed (Figure 7). Thus, allergen-induced asthma-like features must be more aggravated by H-ASD + *B. adusta*.

Addition of OVA to a particle sample consisting of H-ASD and *B. adusta* increased TGF-β (Figure 8). TGF-β1 is well known to possess various biological activities including immunosuppression [26]. However, the suppressive effects on the Th2 response as result of an increase of TGF-β was not detected in this study.

In addition, TGF-β1 is also well known as a repair and profibrotic cytokine [27]. Hyperplasia of bronchial structural cells, like fibroblasts and smooth muscle cells in the airway, is a typical feature of airway remodeling. The induction levels of TGF-β1 in the present study paralleled the severity of fibrous thickening of the subepithelial layer in the airway. Therefore, TGF-β1 may play an important role in the development of serious fibrous thickening (airway remodeling) in this experimental asthma, especially in the OVA + H-ASD + *B.ad* 8 treated groups.

IL-10 produced by Treg cells or Type 1 regulatory T (Tr1) cells also suppresses the Th2 cell-driven response to allergens, as does TGF-β1 [28,29]. However, the suppressive effect to Th2 response by an increase of IL-10 in the OVA + *B.ad* 8 and OVA + H-ASD + *B.ad* 8 treated groups was not detected.

In the present study, an adjuvant effect of *B. adusta* and H-ASD toward OVA-specific IgG1 production was detected but OVA-specific IgE production was not (Figure 9). Antigen-specific IgG1 can cause degranulation via an Fcg RII receptor on the eosinophil’s surface [30]. Therefore, antibodies may play an important role in the aggravation of lung eosinophilia caused by OVA + H-ASD + *B.ad*.

## Conclusions

*B. adusta* isolated from ASD aerosol caused murine lung eosinophilia and heat-treated ASD (H-ASD) aggravated the lung eosinophilia associated with *B. adusta. B. adusta* aggravated OVA-induced lung eosinophilia. A mixture of *B. adusta*, H-ASD, and OVA caused the most remarkable exacerbation to the allergic airway inflammation in experimental animals. Therefore, the present study indicates that *B. adusta* is an allergic exacerbating factor and acts as an inducer of eosinophilic lung disease and that H-ASD is an allergic exacerbating factor.

The adverse effects of dust containing minerals and biogenic agents on human respiratory diseases have become a public concern. In particular, atmospheric exposure to fungi, bacteria, and silica-carrying particulate matters may affect human health directly through allergic induction of respiratory stress. Therefore, the results obtained in the present study may serve as a warning about the ill effects of airborne sand dust on the human respiratory system and also be useful in finding the appropriate method for treating patients with FACC or asthma. In order to clarify an association of ASD events with the exacerbation of FACC or asthma, epidemiological study is in order.

## Abbreviations

ASD: Asian sand dust; B. adusta: Bjerkandera adusta; OVA: Ovalbumin; BALF: Bronchoalveolar lavage fluid; ELISA: Enzyme-linked immunosorbent assays; FACC: Fungi in fungus-associated chronic cough; H-ASD: Heated Asian sand dust; IFN-γ: Interferon-γ; IL: Interleukin; KC: Keratinocyte chemoattractant; MCP-1: Monocyte chemotactic protein-1; MCP-3: Monocyte chemotactic protein-3; MIP-1α: Macrophage inflammatory protein-1α; NF-κB: Nuclear factor-kappa B; RANTES: Regulated on activation normal T cell expressed and presumably secreted; TGF-β1: Transforming growth factor-β1; TLR: Toll like receptor; TNF-α: Tumor necrosis factor-α.

## Competing interests

The authors do not have any conflicts of interest to disclose.

## Authors’ contribution

TI designed the research. BL, MH, FK, MT, SY, YY, KA, HT, and MN conducted the experiments. TI and TS analyzed the data and wrote the manuscript. TI and GS had primary responsibility for final content. All authors read and approved the final manuscript.
